# Effect of Prosocial Behaviors on e-Consultations in a Web-Based Health Care Community: Panel Data Analysis

**DOI:** 10.2196/52646

**Published:** 2024-04-25

**Authors:** Xiaoxiao Liu, Huijing Guo, Le Wang, Mingye Hu, Yichan Wei, Fei Liu, Xifu Wang

**Affiliations:** 1 School of Management Xi’an Jiaotong University Xi'an China; 2 China Institute of Hospital Development and Reform Xi'an Jiaotong University Xi'an China; 3 School of Economics and Management China University of Mining and Technology Xuzhou China; 4 College of Business City University of Hong Kong Hong Kong China (Hong Kong); 5 School of Economics and Management Xi’an University of Technology Xi'an China; 6 School of Management Harbin Engineering University Harbin China; 7 Healthcare Simulation Center Guangzhou First People’s Hospital Guangzhou China

**Keywords:** prosocial behaviors, proactive behaviors, reactive behaviors, reputations, e-consultation volume, live streaming

## Abstract

**Background:**

Patients using web-based health care communities for e-consultation services have the option to choose their service providers from an extensive digital market. To stand out in this crowded field, doctors in web-based health care communities often engage in prosocial behaviors, such as proactive and reactive actions, to attract more users. However, the effect of these behaviors on the volume of e-consultations remains unclear and warrants further exploration.

**Objective:**

This study investigates the impact of various prosocial behaviors on doctors’ e-consultation volume in web-based health care communities and the moderating effects of doctors’ digital and offline reputations.

**Methods:**

A panel data set containing information on 2880 doctors over a 22-month period was obtained from one of the largest web-based health care communities in China. Data analysis was conducted using a 2-way fixed effects model with robust clustered SEs. A series of robustness checks were also performed, including alternative measurements of independent variables and estimation methods.

**Results:**

Results indicated that both types of doctors’ prosocial behaviors, namely, proactive and reactive actions, positively impacted their e-consultation volume. In terms of the moderating effects of external reputation, doctors’ offline professional titles were found to negatively moderate the relationship between their proactive behaviors and their e-consultation volume. However, these titles did not significantly affect the relationship between doctors’ reactive behaviors and their e-consultation volume (*P*=.45). Additionally, doctors’ digital recommendations from patients negatively moderated both the relationship between doctors’ proactive behaviors and e-consultation volume and the relationship between doctors’ reactive behaviors and e-consultation volume.

**Conclusions:**

Drawing upon functional motives theory and social exchange theory, this study categorizes doctors’ prosocial behaviors into proactive and reactive actions. It provides empirical evidence that prosocial behaviors can lead to an increase in e-consultation volume. This study also illuminates the moderating roles doctors’ digital and offline reputations play in the relationships between prosocial behaviors and e-consultation volume.

## Introduction

### Background

e-Consultations, offered through web-based health care communities [1], are increasingly becoming vital complements to traditional hospital services [2-4]. In hospital consultations, patients can only passively accept treatment [5] from a limited pool of medical resources within a geographical radius. However, when engaging with web-based health care communities, patients can search for primary care solutions [6] from an extensive digital market in a relatively short time [7]. Given that the diagnostic accuracy of e-consultations matches that of hospital consultations [8-10], e-consultations are becoming increasingly attractive to patients [3,11].

Doctors are also showing a growing interest in e-consultations, motivated by economic and social benefits. First, doctors can achieve economic gains by participating in e-consultations [7,12]. Web-based consultation platforms facilitate an efficient reputation system, enabling patients to easily provide feedback about doctors. Consequently, doctors can use e-consultation to strengthen their relationship with patients [13,14] and foster positive word-of-mouth [15]. More e-consultations can benefit doctors by retaining current patients, attracting new ones, and boosting in-person hospital visits [16,17]. Second, doctors could also receive social returns from engaging in e-consultation [7]. Active participation in e-consultations allows doctors to demonstrate their skills, attitude, and experience, aiding in accumulating professional capital [7], building their reputation [18], and increasing their social influence [19]. Given these tangible and intangible benefits, it is essential for doctors to diligently provide the desired e-consultations and make additional efforts to highlight their service attributes to stand out [6,20,21]. This involves engaging in prosocial behaviors in web-based health care communities, which is the primary research focus of this study.

Prior studies have examined the effects of prosocial behaviors on financial outcomes, such as actions reflecting social responsibility in the workplace [22]. In the health care sector, previous research has explored doctors’ prosocial behaviors within traditional, offline medical services. Doctors, working in established medical institutes and serving patients with limited choices of clinical service providers, often aim for self-satisfaction and patient satisfaction with their offline prosocial behaviors. For example, research indicates that doctors may act prosocially to regulate their self-oriented feelings [23] and foster a caring and understanding attitude toward patients [24,25]. Additionally, doctors who demonstrate more empathy and care can elicit positive emotions in patients and improve the doctor-patient relationship [26,27].

Compared to the offline context, doctors’ prosocial behaviors in a digital context may differ in 2 aspects. First, the internet allows patients to choose from a broader, more diverse range of doctors without the constraints of time and space [7]. However, the uncertainty inherent in the digital environment creates a more pronounced information asymmetry between patients and doctors [28], consequently making it more challenging for patients to establish trust. Therefore, doctors’ prosocial behaviors are crucial in building their self-image, establishing patients’ trust, and assisting patients in identifying suitable doctors [29,30]. Second, unlike offline environments, web-based medical platforms offer a range of functions, including asynchronous activities such as publishing articles, as well as real-time interactional actions such as answering questions during live streams. This array of functions facilitates the adoption of more diverse prosocial behaviors by doctors.

Although these differences underscore the importance of studying doctors’ prosocial behavior, there has been limited research focusing on the impact of such behaviors in the digital context. One previous study has scrutinized the impact of prosocial behaviors, such as answering patients’ questions freely, on patient engagement within web-based health care communities [31]. An aspect that requires further exploration is how doctors’ motivations and patients’ involvement vary in doctors’ helping behaviors. Consequently, studies on web-based health care communities should differentiate between diverse prosocial actions to understand their effects on doctors’ web-based service outcomes. This study aims to contribute new knowledge regarding the full breadth of doctors’ prosocial behaviors.

Unlike the previous study that exclusively investigated doctors’ asynchronous behaviors in web-based health care communities [31], this study also explores the role of synchronous reactive actions in achieving optimal doctors’ e-consultation volume. Recently, web-based health care communities have developed and released live-streaming functions to assist doctors in providing voluntary interactions with patients. The effect of doctors’ engagement in medical live streaming on e-consultation services remains unexplored. While these behaviors could demonstrate doctors’ ethical traits and ability to fulfill an e-consultation workflow, a potential trade-off with e-consultations may exist when doctors engage in prosocial behaviors.

In summary, this study examines the effects of doctors’ proactive and reactive prosocial behaviors, considering their digital and offline reputations as potential moderating factors. First, drawing from functional motives theory (FMT), we explore the impact of doctors’ web-based proactive actions on their e-consultation volume. Proactive behaviors are actions in which individuals exceed their assigned work, focusing on long-term goals to prevent future problems [32,33]. According to FMT, these behaviors reflect helping actions that satisfy personal needs [34], driven by self-focused motivations [35], such as impression management and the realization of self-worth goals. For example, knowledge-based proactive behaviors, such as disseminating expertise to preempt future issues, are self-initiated and not reactions to immediate requests [36]. This study categorizes doctors’ sharing of professional articles as a form of proactive behavior that creates a professional image for their patient audience. This is because these actions aim to assist patients with future health concerns rather than directly responding to patients’ immediate needs.

Second, this study explores the role of doctors’ reactive prosocial behaviors in increasing e-consultations, guided by social exchange theory (SET). Unlike proactive behaviors, reactive behaviors are characterized by instances of individuals engaging in helping activities [35], typically in response to others’ needs [34]. SET posits that individuals incurring additional social costs in relationships may anticipate reciprocal value [37,38]. Reactive prosocial behaviors, per SET, are initiated by the motivation to satisfy others’ desires, leading to the development of cooperative social values. In our context, medical live streams facilitate real-time, synchronized interactions, enabling patients to ask questions and doctors to provide immediate responses. Patients’ health questions during these streams indicate their immediate needs. Thus, a higher frequency of live streams within a certain period suggests doctors are increasingly responding to patients’ needs during that time. Therefore, this study uses the number of medical live-streaming sessions conducted by doctors as a measure for their synchronous reactive behaviors.

Finally, considering that doctors’ reputations play a crucial role in their workflow on web-based health care communities [39,40], we test the moderating roles of digital and offline reputation—measured by doctors’ offline professional titles and patients’ recommendations in the digital context, respectively—on the main effects.

### Objective

Based on previous studies and practices within web-based health care communities, we aim to extend the literature by testing the impact of 2 types of web-based prosocial behaviors by doctors: proactive and synchronous reactive actions on e-consultation volume. We then explore the moderating roles of doctors’ offline and digital reputations on these main effects.

### Research Framework and Hypothesis Development

#### Overview

**Figure 1:**
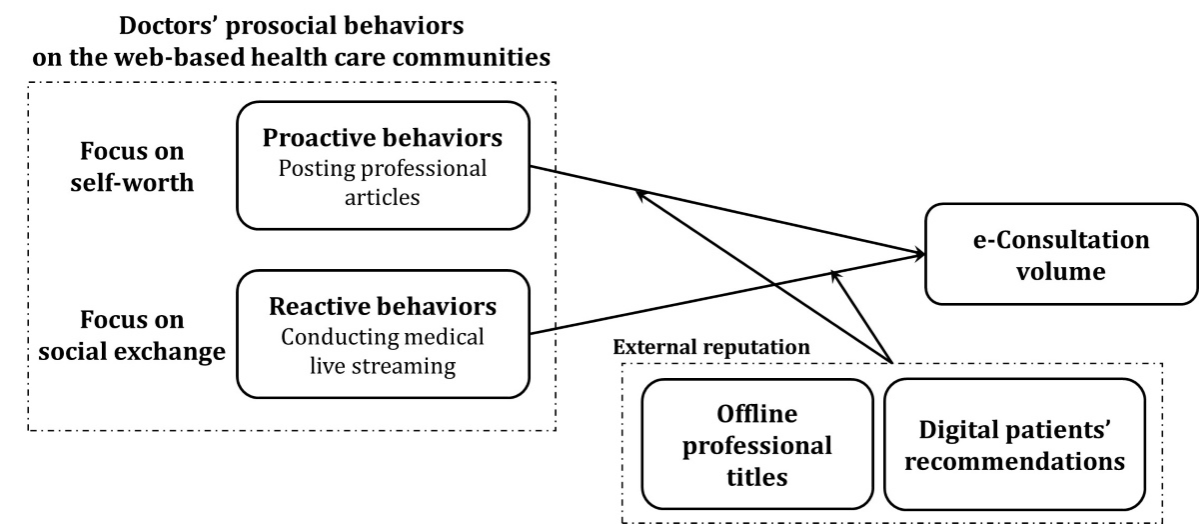
Research framework.

We have developed a research framework, shown in Figure 1, to identify effective prosocial strategies used by doctors within web-based health care communities to achieve a preferred e-consultation volume from the supply side.

Primarily, we explore the relationships between doctors’ prosocial behaviors and e-consultation volume, drawing on FMT and SET. These theories are widely adopted for measuring and classifying the outcomes of prosocial behaviors from 2 fundamental perspectives based on human nature [34]. While doctors’ offline prosocial behaviors may help satisfy patients [24,25], who are already service acceptors, the outcomes of doctors’ web-based prosocial behaviors still need careful distinction. It is essential to clearly differentiate between various types of doctors’ prosocial behaviors to identify their nature. In this study, following the leads of FMT and SET, we test 2 kinds of prosocial behavior: proactive (posting professional articles to achieve self-worth) and reactive (conducting medical live streaming to create cooperative social values).

Subsequently, we examine how doctors’ external reputation moderates the impacts of doctors’ proactive and reactive prosocial behaviors. This examination is conducted from the perspectives of reducing uncertainty and building trust, respectively.

#### Doctors’ Proactive Behaviors and e-Consultation Volume

FMT places emphasis on the primary motivations behind individuals’ behaviors, adopting an atheoretical stance [41]. Through the exploratory process, previous studies have provided examples to identify the functional motivations behind prosocial behaviors [42], such as expressing important personal values. In web-based health care communities, doctors have the opportunity to demonstrate personal traits through proactive behaviors. According to FMT, these proactive behaviors stem from the actors’ active efforts to satisfy their own needs and achieve self-worth [34,35].

Doctors might post professional articles, such as clinical notes and scientific papers, on web-based health care communities to help patient readers handle future health problems. These proactive prosocial behaviors are primarily driven by a desire to showcase personal medical competence, a crucial characteristic of a professional image [43], in medical consultations. By posting professional articles, doctors can display their medical knowledge, care delivery capability, and service quality, thereby enhancing their professional image. We hypothesize that this effort will lead to an increase in the e-consultation volume. Therefore, we propose the following hypothesis:

- Hypothesis 1: The posting of professional articles by doctors positively impacts their e-consultation volume on web-based health care communities.

#### Doctors’ Reactive Behaviors and e-Consultation Volume

Considering the social environment in the working context, SET suggests that reactive prosocial behaviors stem from responding to others’ needs [34]. Engaging in such behaviors can foster positive perceptions among the audience and build cooperative social values [44] through reactive social exchange. People with a high orientation toward cooperative social values act to maximize mutual interests [45], a trait highly valued in the medical field.

We use medical live streaming as a measure of doctors’ reactive behaviors on web-based health care communities. Volunteering to provide interactional live streaming, a typical reactive behavior that may generate cooperative social value, gives the patient audience the impression that the doctors will prioritize demand-side interests during e-consultation services. Additionally, engaging in medical live streaming allows doctors to present themselves as authentic and recognized experts. This enhances their social presence [46], potentially leading to increased service use [47] and greater popularity [48]. Consequently, patients are more likely to perceive doctors who participate in medical live streaming as trustworthy for consultations. Given that e-consultations are closely related to the health conditions of the demand side, a credible doctor is likely to attract more e-consultations. Therefore, we propose the following hypothesis:

- Hypothesis 2: The conduct of medical live streaming by doctors positively impacts their e-consultation volume on web-based health care communities.

#### Moderating Roles of Offline and Digital Reputation

As doctors’ proactive and reactive behaviors potentially affect their consultation performance, based on 2 distinct theoretical foundations of human nature, there exists a discrepancy in how doctors’ reputations influence the relationship between various prosocial behaviors and e-consultation.

We formulate hypotheses regarding the moderating effects within the context of digital health care, by taking into account the inherent information asymmetry and the significance of establishing patient trust. Specifically, our hypotheses explore the influence of reputation on the relationship between doctors’ proactive behaviors and e-consultation volume, with a focus on reducing uncertainty. Additionally, we examine how reputation moderates the impact of doctors’ reactive behaviors, emphasizing the perspective of trust building.

First, in the marketing literature, service providers’ reputations, which can reduce information asymmetry and purchase uncertainty [49], are key factors influencing purchasing behavior and sales performance in the digital context [50-52]. Similarly, for doctors, reputations are related to the experiences and beliefs of other stakeholders [53]. As health care services are credence goods [54]—whose quality patients cannot discern even after experiencing the services—and given the nature of web-based platforms (eg, the absence of face-to-face meetings), there is a significant information asymmetry [51]. This increases patients’ uncertainty regarding the quality of doctors. Consequently, doctors’ reputations play crucial roles in patients’ decision-making processes [18,39]. We use doctors’ professional titles and patients’ recommendations on web-based health care communities to measure doctors’ offline and digital reputations.

Proactive behaviors by low-reputation doctors can create deeper professional impressions [34,35] to reduce uncertainty in e-consultations than high-reputation doctors, who are less uncertain in medical services. Then, doctors’ reputations—measured by offline professional titles and digital patients’ recommendations on web-based health care communities—will negatively moderate the relationship between proactive behavior and e-consultation volume. Thus, we propose the following hypotheses:

- Hypothesis 3a: Doctors’ offline professional titles negatively moderate the relationship between the posting of professional articles and e-consultation volume on web-based health care communities.
- Hypothesis 3b: Doctors’ digital recommendations from patients negatively moderate the relationship between the posting of professional articles and e-consultation volume on web-based health care communities.

Second, one of the central elements of SET is the concept of trust between actors in the exchange process [55-58]. In the context of digital health, patient’s trust in doctors is important to establish in order to refine the doctor-patient relationship. Doctors’ reputations can reflect their personality traits [39] and promote trust from patients [53]. Conducting medical live streaming, a form of reactive prosocial behavior, includes doctors’ cooperative social value orientations that are preferred in e-consultations. For low-reputation doctors, such as those with relatively junior professional titles and few digital patient recommendations, conducting medical live streaming will build patients’ confidence in e-consultations to a greater extent than doctors with high reputations, who are usually already highly trusted. Then, offline and digital reputation may negatively moderate the relationship between engaging in medical live streaming and e-consultation volume. Thus, we propose the final hypotheses:

- Hypothesis 4a: Doctors’ offline professional titles will negatively moderate the relationship between conducting medical live streaming and e-consultation volume on web-based health care communities.
- Hypothesis 4b: Doctors’ digital recommendations from patients will negatively moderate the relationship between conducting medical live streaming and e-consultation volume on web-based health care communities.

## Methods

### Research Context and Data Collection

Our research context is one of the largest web-based health care communities in China. This platform, established in 2006, offers e-consultation services to patients. As of July 2023, it boasts over 260,000 active doctors from 10,000 hospitals nationwide and has provided web-based medical services to 79 million patients.

The platform allows doctors to create home pages where they can display relevant information such as offline professional titles, experiences shared by other patients, and personal introductions. Patients can select doctors for e-consultation by browsing this information. Besides e-consultation, doctors can engage in prosocial behavior primarily focused on knowledge sharing. This includes posting professional articles in various formats (text, voice, and short videos) and conducting medical live streams for real-time interaction with patients.

We collected data over a 22-month period, from January 2021 to October 2022, focusing on common diseases such as diabetes, depression, infertility, skin diseases, and gynecological diseases. To ensure that our findings are generalizable to a typical and active doctor on the platform, we included doctors who had posted at least 1 article and conducted at least 1 live stream before the end of the study period in our analysis [59-61]. Our sample consists of 2880 doctors and includes the following information for each doctor: professional title, patient recommendations, records of experiences shared by the doctor’s patients, records of professional articles posted, records of live streams conducted, and records of the doctor’s e-consultations.

### Variable Operationalization

#### Overview

Our unit of analysis is each doctor. We investigate how doctors’ prosocial behaviors, including proactive behaviors (posting professional articles) and reactive behaviors (conducting medical live streams), influence their e-consultation volume.

#### Dependent Variable

Our dependent variable is the doctors’ e-consultation volume, denoted as *Consultation_it_*, which is measured by the number of e-consultations of doctor *i* in month *t*.

#### Independent Variables

Our independent variables are doctors’ proactive behaviors and reactive behaviors. Doctors’ proactive behavior is operationalized as the posting of professional articles. Specifically, we denote proactive behavior as *Articles_it_*, which is measured by the number of professional articles posted by doctor *i* in month *t*. Doctors’ reactive behavior is operationalized as medical live streaming. This variable is denoted as *LiveStreaming_it_*, which is calculated as the number of medical live streams conducted by doctor *i* in month *t*.

#### Moderating Variables

We are also interested in how doctors’ external reputation, including their offline professional titles and digital recommendations from patients, influences the relationship between prosocial behaviors and e-consultation volume. A doctor’s offline professional title is denoted as *Title_i_*, which is a dummy variable indicating whether doctor *i* is a chief doctor (*Title_i_*=1 indicates the doctor is a chief doctor, and *Title_i_*=0 indicates the doctor has a lower-ranked title). Digital recommendations are captured by *Recommendations_i_*, which is the digital recommendation level of doctor *i* as calculated by the platform based on the recommendations provided by their past patients.

#### Control Variables

We incorporated several control variables to account for factors that may influence patient’s choices of doctors in the digital context. The shared experiences of patients regarding a doctor’s treatment [39], as well as the number of patients who have previously consulted with the doctors [17,62], can indicate the doctor’s overall popularity. This, in turn, may affect patient choice. Therefore, we controlled for (1) the total number of patients who consulted with doctor *i* in the digital context before month *t* (*TotalPatients_it_*) and (2) the total number of patient-shared experiences about offline treatment by doctor *i* before month *t* (*TotalExperiences_it_*). Furthermore, doctors’ past behaviors, including article publishing and live streaming, can influence their current practices in posting articles and conducting live streams. Simultaneously, these factors may also act as signals affecting patients’ judgments and selection of doctors [12]. To account for these influences, we also controlled for (1) the total number of articles posted by doctor *i* before month *t* (*TotalArticles_it_*) and (2) the total number of medical live streams conducted by doctor *i* before month *t* (*TotalLiveStreaming_it_*).

To control for both observed and unobserved doctor-specific factors that do not change over time, individual-fixed effects were added. Additionally, time-fixed effects were introduced into our analysis to account for both observed and unobserved factors that vary over time but remain constant across doctors. Table 1 shows the variables and their definitions.

**Table 1:** Variable definition.

Variables		Definition
**Dependent variable**		
	*Consultation_it_*	The number of e-consultations of doctor *i* in month *t*.
**Independent variables**		
	*Articles_it_*	The number of professional articles posted by doctor *i* in month *t*.
	*LiveStreaming_it_*	The number of medical live streams conducted by doctor *i* in month *t*.
**Moderating variables**		
	*Title_i_*	A dummy variable indicating whether doctor *i* is a chief doctor (takes the value 1 for a chief doctor and 0 for doctors with lower-ranked titles).
	*Recommendations_i_*	The digital recommendation level of doctor *i* by other patients.
**Control variables**		
	*TotalPatients_it_*	The total number of patients who consulted doctor *i* in the digital context before month *t*.
	*TotalExperiences_it_*	The total number of patient-shared experiences about offline treatment by doctor *i* before month *t*.
	*TotalArticles_it_*	The total number of articles posted by doctor *i* before month *t*.
	*TotalLiveStreaming_it_*	The total number of medical live streams doctor *i* conducted before month *t*.

### Estimation Model

To estimate the direct impact of doctors’ proactive behaviors and reactive behaviors on their e-consultation volume, the following 2-way fixed effects regression model was used:

*Consultation_it_* = *β*_0_ + *β*_1_*Articles_it_* + *β*_2_*LiveStreaming_it_* + *β*_3_*TotalPatients_it_* + *β*_4_*TotalExperiences_it_* + *β*_5_*TotalArticles_it_* + *β*_6_*TotalLiveStreaming_it_* + *α_i_* + *δ_t_* + *μ_it_*
**(1)**

where *i* denotes doctor, *t* denotes month, *α_i_* is doctor-fixed effects, *δ_t_* is month-fixed effects, *Consultation_it_* is the number of e-consultations of doctor *i* in month *t*, *Articles_it_* is the number of professional articles posted by doctor *i* in month *t*, *LiveStreaming_it_* is the number of medical live streams conducted by doctor *i* in month *t*, *TotalPatients_it_* is the total number of patients who consulted doctor *i* in the digital context before month *t*, *TotalExperiences_it_* is the total number of patient-shared experiences about offline treatment by doctor *i* before month *t*, *TotalArticles_it_* is the total number of articles posted by doctor *i* before month *t*, *TotalLiveStreaming_it_* is the total number of medical live streams doctor *i* conducted before month *t*, *β* is the coefficient, and *μ_it_* is the error term. We took the log transformation for our continuous variables in the model to reduce the skewness of the variables [63].

Next, the moderating effects of doctors’ offline professional titles and digital recommendations by patients were investigated based on the following specification:

*Consultation_it_* = *β*_0_ + *β*_1_*Articles_it_* + *β*_2_*LiveStreaming_it_* + *β*_3_*Articles_it_* × *Title_i_* + *β*_4_*LiveStreaming_it_* × *Title_i_* + *β*_5_*Articles_it_* × *Recommendation_i_* + *β*_6_*LiveStreaming_it_* × *Recommendation_i_* + *β*_7_*TotalPatients_it_* + *β*_8_*TotalExperiences_it_* +*β*_9_*TotalArticles_it_* + *β*_10_*TotalLiveStreaming_it_* + *α_i_* + *δ_t_* + *μ_it_*
**(2)**

where *Title_i_* indicates whether doctor *i* is a chief doctor (*Title_i_*=1 indicates the doctor is a chief doctor, and *Title_i_*=0 indicates the doctor has a lower-ranked title). *Recommendations_i_* is the digital recommendation level of doctor *i* by other patients.

### Ethical Considerations

This study used secondary publicly available data obtained from a website and did not involve the collection of original data pertaining to human participants. As such, there is no evidence of unethical behavior in the study. Consequently, ethics approval by an ethics committee or institutional review board was not deemed necessary.

## Results

### Overview

In this section, we present our empirical results. The descriptive statistics are shown in Table 2, and the correlation matrix is shown in Table 3.

**Table 2:** Descriptive statistics.

Variable	Mean (SD)	Minimum-maximum
*Consultation*	17.529 (41.172)	0-1148
*Articles*	1.478 (8.385)	0-658
*LiveStreaming*	0.196 (0.928)	0-39
*Title*	0.413 (0.492)	0-1
*Recommendations*	3.849 (0.518)	1.5-5
*TotalPatients*	1469.475 (3237.306)	0-72,559
*TotalExperiences*	209.578 (363.915)	0-4626
*TotalArticles*	59.552 (203.213)	0-5795
*TotalLiveStreaming*	2.313 (7.171)	0-163

**Table 3:** Correlation matrix.

Variable			*Consultation*		*Articles*		*LiveStreaming*		*Title*		*Recommendations*		*TotalPatients*		*TotalExperiences*		*TotalArticles*		*TotalLiveStreaming*
***Consultation***																			
	*r*	1		0.177		0.146		0.132		0.561		0.629		0.628		0.355		0.192	
	*P* value	—		<.001		<.001		<.001		<.001		<.001		<.001		<.001		<.001	
***Articles***																			
	*r*	0.177		1		0.39		0.018		0.156		0.097		0.117		0.353		0.233	
	*P* value	<.001		—		<.001		<.001		<.001		<.001		<.001		<.001		<.001	
***LiveStreaming***																			
	*r*	0.146		0.390		1		0.006		0.072		0.066		0.071		0.176		0.348	
	*P* value	<.001		<.001		—		.14		<.001		<.001		<.001		<.001		<.001	
***Title***																			
	*r*	0.132		0.018		0.006		1		0.203		0.238		0.248		0.174		0.014	
	*P* value	<.001		<.001		.14		—		<.001		<.001		<.001		<.001		<.001	
***Recommendations***																			
	*r*	0.561		0.156		0.072		0.203		1		0.42		0.648		0.304		0.091	
	*P* value	<.001		<.001		<.001		<.001		—		<.001		<.001		<.001		<.001	
***TotalPatients***																			
	*r*	0.629		0.097		0.066		0.238		0.420		1		0.774		0.569		0.245	
	*P* value	<.001		<.001		<.001		<.001		<.001		—		<.001		<.001		<.001	
***TotalExperiences***																			
	*r*	0.628		0.117		0.071		0.248		0.648		0.774		1		0.513		0.208	
	*P* value	<.001		<.001		<.001		<.001		<.001		<.001		—		<.001		<.001	
***TotalArticles***																			
	*r*	0.355		0.353		0.176		0.174		0.304		0.569		0.513		1		0.415	
	*P* value	<.001		<.001		<.001		<.001		<.001		<.001		<.001		—		<.001	
***TotalLiveStreaming***																			
	*r*	0.192		0.233		0.348		0.014		0.091		0.245		0.208		0.415		1	
	*P* value	<.001		<.001		<.001		<.001		<.001		<.001		<.001		<.001		—	

### Empirical Results

#### Results for Direct Effects

The analysis was conducted progressively. We first estimated the equation without control variables (model 1) and then added control variables in model 2. The estimated results are shown in Table 4. From the results, we can see that the coefficient of *Articles* is significant and positive in model 2 (β=.093; *P*<.001), indicating that doctors’ proactive behaviors (ie, posting professional articles) can help them obtain more e-consultations. Thus, hypothesis 1 is supported. Regarding doctors’ engagement in medical live streaming, the results show that the coefficient of *LiveStreaming* is significantly positive (β=.214; *P*<.001), which suggests that doctors’ reactive behaviors (ie, conducting medical live streaming) can increase their e-consultation volume. This supports hypothesis 2.

**Table 4:** The effects of doctors’ proactive behaviors and reactive behaviors on their e-consultation volume^a^.

Variables	e-Consultation volume: *Consultation*			
	Model 1^b^		Model 2^c^	
	β (SE)	*P* value	β (SE)	*P* value
*Articles*	.096 (0.008)	<.001	.093 (0.008)	<.001
*LiveStreaming*	.235 (0.016)	<.001	.214 (0.016)	<.001
*TotalPatients*	N/A^d^	N/A	.195 (0.027)	<.001
*TotalExperiences*	N/A	N/A	.256 (0.037)	<.001
*TotalArticles*	N/A	N/A	.073 (0.014)	<.001
*TotalLiveStreaming*	N/A	N/A	–.003 (0.015)	.81
*Constant*	1.704 (0.003)	<.001	–.617 (0.120)	<.001

^a^All models include doctor-fixed effects and month-fixed effects; robust SEs clustered by doctors are reported; the number of doctors is 2880, and the number of observations is 63,360.

^b^*R*^2^=0.843; *F*_2,2879_=175.98; *P*<.001.

^c^*R*^2^=0.851; *F*_6,2879_=119.72; *P*<.001.

^d^N/A: not applicable.

#### Results for Moderating Effects

The results for moderating effects are shown in Table 5. In model 1, interaction terms were initially introduced between *Title* and *Articles*, as well as between *Title* and *LiveStreaming*, to estimate the moderating effect of doctors’ offline professional titles. The interaction terms were then added between *Recommendations* and *Articles*, as well as between *Recommendations* and *LiveStreaming*, to estimate the moderating effect of doctors’ digital recommendations in model 2. Finally, a full model was estimated by incorporating all interaction terms. We find that the results are consistent across all models. Wald tests and likelihood ratio were used to compare the fit among nested models [64,65], and the results show that the inclusion of moderating variables significantly enhances the model’s fit.

Regarding the moderating effect of doctors’ offline professional titles, we find that the coefficient of *Articles*×*Title* in model 1 of Table 5 is significantly negative (β=–.058; *P*<.001), which supports hypothesis 3a that doctors’ offline professional titles have a negative moderating effect on the relationship between doctors’ proactive behaviors and e-consultation volume. However, the coefficient of *LiveStreaming*×*Title* is insignificant (β=–.024; *P*=.45), which suggests that doctors’ offline professional titles have no moderating effect on the relationship between doctors’ reactive behaviors and e-consultation volume. Thus, hypothesis 4a is not supported.

**Table 5:** The moderating effects of doctors’ offline professional titles and digital patient recommendations^a^.

Variables	e-Consultation volume: *Consultation*					
	Model 1^b^		Model 2^c^		Model 3^d^	
	β (SE)	*P* value	β (SE)	*P* value	β (SE)	*P* value
*Articles*	.117 (0.011)	<.001	.313 (0.057)	<.001	.303 (0.057)	<.001
*LiveStreaming*	.223 (0.022)	<.001	.603 (0.114)	<.001	.601 (0.112)	<.001
*Articles*×*Title*	–.058 (0.016)	<.001	N/A^e^	N/A	–.047 (0.016)	.003
*LiveStreaming*×*Title*	–.024 (0.031)	.45	N/A	N/A	.007 (0.031)	.83
*Articles*×*Recommendations*	N/A	N/A	–.055 (0.014)	<.001	–.048 (0.014)	<.001
*LiveStreaming*×*Recommendations*	N/A	N/A	–.100 (0.028)	<.001	–.100 (0.027)	<.001
*TotalPatients*	.193 (0.027)	<.001	.193 (0.027)	<.001	.193 (0.027)	<.001
*TotalExperiences*	.259 (0.037)	<.001	.262 (0.037)	<.001	.263 (0.037)	<.001
*TotalArticles*	.071 (0.014)	<.001	.072 (0.014)	<.001	.071 (0.014)	<.001
*TotalLiveStreaming*	–.002 (0.015)	.87	–.002 (0.015)	.88	–.002 (0.015)	.91
*Constant*	–.614 (0.121)	<.001	–.628 (0.121)	<.001	–.625 (0.121)	<.001

^a^All models include doctor-fixed effects and month-fixed effects; robust SEs clustered by doctors are reported; the number of doctors is 2880, and the number of observations is 63,360.

^b^*R*^2^=0.851; *F*_8,2879_=89.98; *P*<.001; Wald test: *P*<.001; likelihood ratio: *P*<.001.

^c^*R*^2^=0.851; *F*_8,2879_=89.13; *P*<.001; Wald test: *P*<.001; likelihood ratio: *P*<.001.

^d^*R*^2^=0.852; *F*_10,2879_=71.44; *P*<.001; Wald test: *P*<.001; likelihood ratio: *P*<.001.

^e^N/A: not applicable.

For the moderating effect of digital patient recommendations, we find that both of the coefficients of *Articles​*×*Recommendations* and *LiveStreaming*×*Recommendations* are negative and significant (β=–.055; *P*<.001 and β=–.100; *P*<.001, respectively, in model 2 of Table 5). This indicates that digital recommendations from patients have negative moderating effects on the relationship between doctors’ proactive behaviors and e-consultation volume as well as on the relationship between doctors’ reactive behaviors and e-consultation volume; this finding supports hypotheses 3b and 4b.

### Robustness Check

First, additional analysis was performed to check whether our findings are robust to different measures of doctors’ reactive behaviors. In the main analysis, we used the number of medical live streams to construct doctors’ reactive behaviors. In the robustness check, doctors’ reactive behaviors were measured using the following measures: (1) the length of time spent in medical live streaming (*LSDuration_it_*), which is calculated as the total duration of all medical live streams conducted by doctor *i* in month *t*; and (2) the number of doctor-patient interactions in the medical live streams (*LSInteractions_it_*), which is calculated as the total number of interactions between doctor *i* and patients in medical live streams in month *t*. This measure is likely to more effectively capture the reactive element of the behavior. The estimated results are shown in Table 6, and we can see that the results are consistent with the main results.

Second, in the above analysis, the total number of articles posted by the doctors was used to measure doctors’ proactive behaviors. As doctors can post articles that are either their own original work or reposts from others, we further used the number of original articles (*OriArticles_it_*) to measure doctors’ proactive prosocial behaviors. Specifically, the number of articles was replaced with the number of original articles posted by doctor *i* in month *t* (*OriArticles_it_*). Models 1 and 2 in Table 7 show the results. We can see that using this alternative measure of proactive behavior does not materially change the results.

Third, as our dependent variable takes nonnegative values, negative binomial regression was further used to re-estimate our models. We find that the results (models 3 and 4 in Table 7) are similar to the main results.

Fourth, to further enhance the robustness and validity of our findings, article quality was used as a measure of doctors’ proactive behaviors. This approach is based on the premise that article quality more accurately reflects the effort and time invested by doctors in content creation. Specifically, we assessed article quality based on either the length of each article or the number of likes it received and then re-estimated our model. As indicated in Table 8, the results remain consistent with our main findings, thereby further reinforcing the validity of our conclusions.

**Table 6:** Robustness checks: alternative measure of reactive behaviors^a^.

Variables	e-Consultation volume: *Consultation*										
	Use *LSDuration* to measure reactive behaviors						Use *LSInteractions* to measure reactive behaviors				
	Model 1^b^		Model 2^c^			Model 3^d^				Model 4^e^	
	β (SE)	*P* value	β (SE)	*P* value	β (SE)			*P* value	β (SE)		*P* value
*Articles*	.095 (0.008)	<.001	.304 (0.058)	<.001	.103 (0.008)			<.001	.351 (0.059)		<.001
*LSDuration*	.023 (0.002)	<.001	.071 (0.011)	<.001	N/A^f^			N/A	N/A		N/A
*LSInteractions*	N/A	N/A	N/A	N/A	.041 (0.004)			<.001	.114 (0.035)		.001
*Articles*×*Title*	N/A	N/A	–.045 (0.016)	.004	N/A			N/A	–.049 (0.016)		.002
*LSDuration*×*Title*	N/A	N/A	–.000 (0.003)	.96	N/A			N/A	N/A		N/A
*LSInteractions*×*Title*	N/A	N/A	N/A	N/A	N/A			N/A	.003 (0.009)		.69
*Articles*×*Recommendations*	N/A	N/A	–.048 (0.014)	.001	N/A			N/A	–.057 (0.014)		<.001
*LSDuration*×*Recommendations*	N/A	N/A	–.012 (0.003)	<.001	N/A			N/A	N/A		N/A
*LSInteractions*×*Recommendations*	N/A	N/A	N/A	N/A	N/A			N/A	–.019 (0.008)		.02
*TotalPatients*	.194 (0.027)	<.001	.192 (0.027)	<.001	.194 (0.027)			<.001	.191 (0.027)		<.001
*TotalExperiences*	.256 (0.037)	<.001	.263 (0.037)	<.001	.258 (0.038)			<.001	.266 (0.038)		<.001
*TotalArticles*	.073 (0.014)	<.001	.071 (0.014)	<.001	.076 (0.014)			<.001	.074 (0.014)		<.001
*TotalLiveStreaming*	.005 (0.015)	.71	.008 (0.015)	.60	–.005 (0.015)			.73	–.004 (0.015)		.80
*Constant*	–.617 (0.120)	<.001	–.626 (0.121)	<.001	–.621 (0.120)			<.001	–.628 (0.122)		<.001

^a^All models include doctor-fixed effects and month-fixed effects; robust SEs clustered by doctors are reported; the number of doctors is 2880, and the number of observations is 63,360.

^b^*R*^2^=0.851; *F*_6,2879_=131.71; *P*<.001.

^c^*R*^2^=0.851; *F*_10,2879_=79.29; *P*<.001; Wald test: *P*<.001; likelihood ratio: *P*<.001.

^d^*R*^2^=0.850; *F*_6,2879_=112.52; *P*<.001.

^e^*R*^2^=0.851; *F*_10,2879_=68.43; *P*<.001; Wald test: *P*<.001; likelihood ratio: *P*<.001.

^f^N/A: not applicable.

**Table 7:** Robustness checks: alternative measure of proactive behaviors and negative binomial regression^a^.

Variables	e-Consultation volume: *Consultation*								
	Use *OriArticles* to measure proactive behaviors					Negative binomial regression			
	Model 1^b^		Model 2^c^		Model 3^d^			Model 4^e^	
	β (SE)	*P* value	β (SE)	*P* value	β (SE)		*P* value	β (SE)	*P* value
*Articles*	N/A^f^	N/A	N/A	N/A	.040 (0.008)		<.001	.277 (0.058)	<.001
*OriArticles*	.093 (0.008)	<.001	.290 (0.057)	<.001	N/A		N/A	N/A	N/A
*LiveStreaming*	.215 (0.016)	<.001	.626 (0.111)	<.001	.166 (0.013)		<.001	.741 (0.150)	<.001
*Articles*×*Title*	N/A	N/A	N/A	N/A	N/A		N/A	–.040 (0.016)	.010
*OriArticles*×*Title*	N/A	N/A	–.051 (0.016)	.001	N/A		N/A	N/A	N/A
*LiveStreaming*×*Title*	N/A	N/A	.008 (0.031)	.80	N/A		N/A	–.026 (0.031)	.40
*Articles*×*Recommendations*	N/A	N/A	N/A	N/A	N/A		N/A	–.050 (0.013)	<.001
*OriArticles*×*Recommendations*	N/A	N/A	–.044 (0.014)	.001	N/A		N/A	N/A	N/A
*LiveStreaming*×*Recommendations*	N/A	N/A	–.106 (0.027)	<.001	N/A		N/A	–.133 (0.036)	<.001
*TotalPatients*	.195 (0.027)	<.001	.192 (0.027)	<.001	.067 (0.023)		.003	.064 (0.023)	.006
*TotalExperiences*	.256 (0.037)	<.001	.263 (0.037)	<.001	.401 (0.026)		<.001	.412 (0.026)	<.001
*TotalArticles*	.073 (0.014)	<.001	.070 (0.014)	<.001	–.011 (0.018)		.55	–.015 (0.015)	.32
*TotalLiveStreaming*	–.003 (0.015)	.84	–.001 (0.015)	.94	.020 (0.016)		.21	.020 (0.019)	.31
*Constant*	–.614 (0.120)	<.001	–.621 (0.121)	<.001	–1.174 (0.076)		<.001	–1.196 (0.091)	<.001

^a^All models include doctor-fixed effects and month-fixed effects; robust SEs clustered by doctors are reported in models 1 and 2; bootstrap SEs in models 3 and 4.

^b^*R*^2^=0.851; *F*_6,2879_=118.99; *P*<.001.

^c^*R*^2^=0.852; *F*_10,2879_=71.04; *P*<.001; Wald test: *P*<.001; likelihood ratio: *P*<.001.

^d^Log likelihood=–150,015.36.

^e^Log likelihood=–149,888.24.

^f^N/A: not applicable.

**Table 8:** Robustness checks: using article quality to measure proactive behavior^a^.

Variables	e-Consultation volume: *Consultation*								
	Using the number of likes received by each article to measure article quality					Using the length of each article to measure article quality			
	Model 1^b^		Model 2^c^		Model 1^d^			Model 2^e^	
	β (SE)	*P* value	β (SE)	*P* value	β (SE)		*P* value	β (SE)	*P* value
*ArticleQuality*	.084 (0.007)	<.001	.539 (0.060)	<.001	.029 (0.002)		<.001	.117 (0.016)	<.001
*LiveStreaming*	.265 (0.016)	<.001	.770 (0.121)	<.001	.241 (0.016)		<.001	.642 (0.117)	<.001
*ArticleQuality*×*Title*	N/A^f^	N/A	–.059 (0.013)	<.001	N/A		N/A	–.016 (0.004)	<.001
*LiveStreaming*×*Title*	N/A	N/A	–.021 (0.032)	.51	N/A		N/A	–.006 (0.032)	.85
*ArticleQuality*×*Recommendations*	N/A	N/A	–.099 (0.014)	<.001	N/A		N/A	–.020 (0.004)	<.001
*LiveStreaming*×*Recommendations*	N/A	N/A	–.127 (0.029)	<.001	N/A		N/A	–.103 (0.028)	<.001
*TotalPatients*	.194 (0.027)	<.001	.195 (0.027)	<.001	.194 (0.027)		<.001	.192 (0.027)	<.001
*TotalExperiences*	.256 (0.037)	<.001	.259 (0.037)	<.001	.257 (0.037)		<.001	.263 (0.037)	<.001
*TotalArticles*	.077 (0.014)	<.001	.077 (0.014)	<.001	.075 (0.014)		<.001	.074 (0.014)	<.001
*TotalLiveStreaming*	.004 (0.015)	.80	.006 (0.015)	.66	.002 (0.014)		.88	.004 (0.015)	.77
*Constant*	–.622 (0.120)	<.001	–.639 (0.121)	<.001	–.627 (0.120)		<.001	–.638 (0.121)	<.001

^a^All models include doctor-fixed effects and month-fixed effects; robust SEs clustered by doctors are reported; the number of doctors is 2880, and the number of observations is 63,360.

^b^*R*^2^=0.851; *F*_6,2879_=127.75; *P*<.001.

^c^*R*^2^=0.851; *F*_10,2879_=89.97; *P*<.001; Wald test: *P*<.001; likelihood ratio: *P*<.001.

^d^*R*^2^=0.851; *F_6_*_,_*_2879_*=133.39; *P*<.001.

^e^*R*^2^=0.852; *F_10_*_,_*_2879_*=84.94; *P*<.001; Wald test: *P*<.001; likelihood ratio: *P*<.001.

^f^N/A: not applicable.

## Discussion

### Analysis of Results

Web-based medical platforms offer a variety of functions to support doctors’ engagement in different types of prosocial behaviors. However, few studies have investigated the effects of these behaviors. Drawing on FMT and SET, this study categorized doctors’ prosocial practices in web-based health care communities into proactive and reactive actions and examined their effects on e-consultation volume. Briefly, prosocial behaviors positively impact on e-consultation, and a doctor’s digital and offline reputation moderates the relationship between prosocial behavior and e-consultation, albeit with some nuances.

First, we expanded upon existing literature on proactive prosocial behaviors, concluding that these actions can help doctors create professional images [43] in the medical consultation context. Our panel data analysis reveals that doctors’ posting of professional articles, which contribute to their professional image in the digital context, attracts more e-consultations. This finding aligns with the prior study [31], which observed that a health professional’s previous asynchronous prosocial behavior positively influences their future economic performance.

Second, drawing from SET, we analyzed the impact of synchronous reactive prosocial behaviors, a less explored area in prior literature. Our findings confirm that engaging in medical live streaming, a form of reactive prosocial behavior, leads to higher e-consultation volumes. Interestingly, we found that the positive impact of conducting a live stream exceeds that of posting an article.

Third, we expanded our research by testing the moderating roles of digital and offline reputations, measured by doctors’ offline professional titles and patients’ recommendations on web-based health care communities. We found that digital reputations significantly moderate the relationships between both types of prosocial behaviors and e-consultation volume. Specifically, doctors who post professional articles or conduct medical live streams attract more e-consultations when they have fewer patient recommendations compared to those with higher recommendations. Regarding offline professional titles, our results indicate a significant moderating effect on the relationship between proactive prosocial behaviors and e-consultation volume. Notably, junior doctors should focus more on posting articles in web-based health care communities to compensate for limitations associated with their titles [66]. However, the moderating effect of offline titles on the impact of reactive prosocial behaviors was found to be insignificant. We attribute this to the unique dynamics of trust conversion in Chinese health care settings. As doctors’ offline titles are granted by medical institutions, these titles could enhance patients’ trust in doctors only if there is a conversion of trust from the organization to the individual doctor, which represents different types of trust [67]. Consequently, doctors with the same offline titles from different hospitals may be perceived differently. For example, a senior doctor from a 3-A hospital is usually seen as highly professional in their clinical field, while a doctor with the same title in a 1-A hospital might typically handle primary diseases. Due to this trust conversion phenomenon, patients may not uniformly trust doctors from different hospitals with the same offline titles, leading to the insignificant moderating effect of offline titles on the impact of reactive prosocial behaviors.

In summary, this study underscores the importance of prosocial behaviors and reputation in shaping doctors’ e-consultation volumes on web-based health care communities, offering valuable insights for health care professionals aiming to increase their consultation outreach.

### Implications

This study makes several theoretical implications. First, this study contributes to web-based health care community literature by offering a nuanced understanding of how doctors’ prosocial behaviors enhance e-consultation volume. While a limited number of studies have examined the effects of doctors’ freely provided behaviors in the digital context [31], the specific impact of different types of prosocial behaviors on e-consultation volume remains largely unexplored. This study addresses this knowledge gap by theoretically categorizing doctors’ prosocial behaviors in web-based health care communities into proactive and reactive types and exploring their impacts on e-consultations.

Second, this study enriches web-based health care communities and live streaming literature by validating the role of medical live streaming in web-based health services. Prior research on live streaming has mainly concentrated on e-commerce [68], web-based gaming [69], and web-based learning [70]. Our study extends this research to the health care context, highlighting the importance of live streaming on web-based health care platforms. Specifically, this study delves into how doctors’ synchronous, reactive volunteer interactions via live streaming influence patient decision-making.

Finally, this study advances FMT and SET by highlighting the importance of context in theory development and providing guidance for context-specific theorizing on web-based health platforms. It also sheds light on how the impact of different prosocial behaviors on e-consultation volume varies depending on a doctor’s offline and digital reputations. Notably, this study validates that proactive behaviors work more effectively in promoting e-consultations for doctors with lower titles or fewer digital recommendations, while reactive behaviors are more effective for doctors with fewer digital recommendations.

This study offers several practical implications for doctors and platform managers. First, the beneficial effects of prosocial behaviors suggest that doctors should adapt their engagement activities when participating in web-based health care platforms. Nowadays, an increasing number of doctors are joining web-based health care communities and focusing on e-consultations, attracted by the economic and social benefits. Based on our results, posting professional articles can help doctors establish a professional image, potentially leading to more e-consultations. Additionally, conducting medical live streams can bolster e-consultations by fostering cooperative social value for doctors and enhancing their credibility among patient audiences. Therefore, doctors may prefer engaging in both proactive and reactive prosocial activities in web-based health care communities to attract more patients to their e-consultation services.

Second, the boundary conditions of the effects of prosocial behaviors imply that doctors should strategically leverage the beneficial effect of proactive and reactive behaviors according to their offline and digital reputations. Doctors with fewer digital recommendations should focus more on prosocial behavior to attract patients to e-consultations. Meanwhile, doctors with lower titles should devote their efforts to proactive behaviors to demonstrate their capability in fulfilling the e-consultations, thereby reducing information asymmetry between patients and themselves.

Third, our findings offer implications for web-based health care platform managers in designing effective functions. An increasing number of platforms are launching various features to better serve doctors and patients, meeting the needs of both groups more effectively. Our empirical findings suggest that doctors’ proactive and reactive prosocial behaviors, such as posting professional articles and conducting medical live streams, can help them establish professional image and enhance patient trust, leading to improved performance. Importantly, these behaviors also benefit patients by enhancing their health knowledge and literacy. Thus, platform managers could introduce functions (eg, article posting, live streaming, and doctor-driven communities) to encourage more prosocial behaviors by doctors. Additionally, platform managers might consider incorporating guidelines or incentive mechanisms for prosocial behaviors into their platforms. For example, it is recommended that platforms collect and analyze doctors’ proactive and reactive prosocial behaviors and guide them on how to effectively use these functions and engage in different types of activities.

### Limitations

Despite its contributions, this study also presents several limitations that future research should consider. First, various classifications of prosocial behavior are available; for instance, Richaud et al [71] classified such behavior as altruistic, compliant, emotional, public, anonymous, or dire actions. Given the intricacy of web-based medical services, future studies would benefit from further exploring the roles of these other types of prosocial behavior exhibited by doctors on web-based health care communities. Second, our research model was constructed primarily from the doctor’s perspective and thus did not investigate the influence of doctors’ prosocial behaviors on patients’ satisfaction and well-being. Future research should delve into these relationships to obtain a more comprehensive understanding of the impacts of doctors’ prosocial behaviors. Finally, this study focused only on the quantity of medical live-streaming sessions, overlooking the quality aspect, which could be a crucial factor influencing e-consultation volume. Future research will concentrate on exploring this aspect.

### Conclusions

Building upon prior studies on doctors’ prosocial behaviors on web-based health care communities, this study further delineates doctors’ beneficial actions into proactive and synchronous reactive behaviors. This distinction is based on the divergence in doctors’ motives for engaging and patients’ levels of involvement. Drawing from FMT and SET, this study offers insights that could aid doctors in increasing their e-consultation volume by adopting these beneficial behaviors. Concurrently, this research augments our understanding of the roles a doctor’s reputation plays in the relationships between various prosocial behaviors—specifically, proactive and reactive actions—and their e-consultation volume. This study may inspire doctors with comparatively lower offline professional titles and digital popularity to achieve their desired e-consultation volume.

## Abbreviations

FMT: functional motives theory
SET: social exchange theory
